# Supplementary material for: Conditional myeloid-specific deletion of Ube2n hinders YUMM1.7 melanoma growth
Source: Front Oncol. 2026 Sep 24;16:1932270. doi: 10.3389/fonc.2026.1932270 (PMC13645565; doi:10.3389/fonc.2026.1932270)
Supplement: Supplementary file 1 [file DataSheet1.pdf]

Supplementary Materials for:

Conditional Myeloid-Specific Deletion of Ube2n Hinders YUMM1.7 Melanoma Growth

Kelsie Schiavone[1] , Adam Pecoraro[1] , Afsa Khawar[1] , Kathleen Zhang[1] , Vaibhav Jain[2] , Simon Gregory[2] , Daniel T. Starczynowski[3] , and Jennifer Y. Zhang[1,4]*

1 Department of Dermatology, Duke University Medical Center, Durham, NC, USA

2 Duke Molecular Physiology Institute, Durham, NC, USA

3 Division of Experimental Hematology and Cancer Biology, Cincinnati Children's Hospital, Cincinnati, OH, USA

4 Department of Pathology, Duke University Medical Center, Durham, NC, USA

*Corresponding author: Jennifer Zhang, jennifer.zhang@duke.edu

This file includes:

Figure S1.

Figure S2.

Figure S3.

Figure S4.

Figure S5.

Table S1. RT-PCR-Primers

Table S2. Key resources table

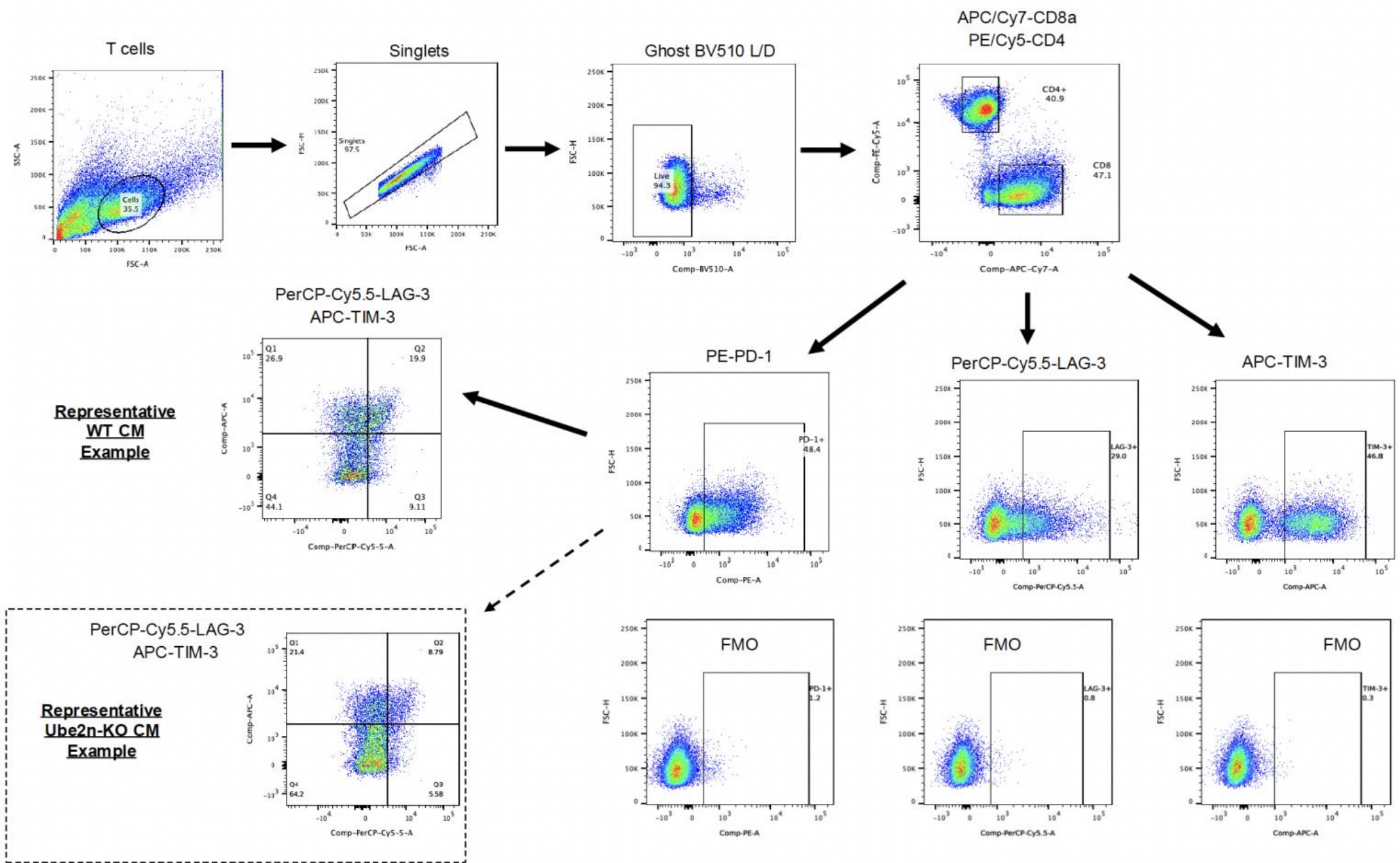

Supplementary Figure S1: Gating strategy for Fig. 4(F), T-cell/BMDM-CM co-culture. T-cell population isolated from debris into singlets, live cells, CD8+ T cells, and PD-1+ LAG-3+, TIM-3+ live single cells as the final gate. FMO gates for PD-1, LAG-3, and TIM-3 are represented alongside representative samples across genotypes tested.

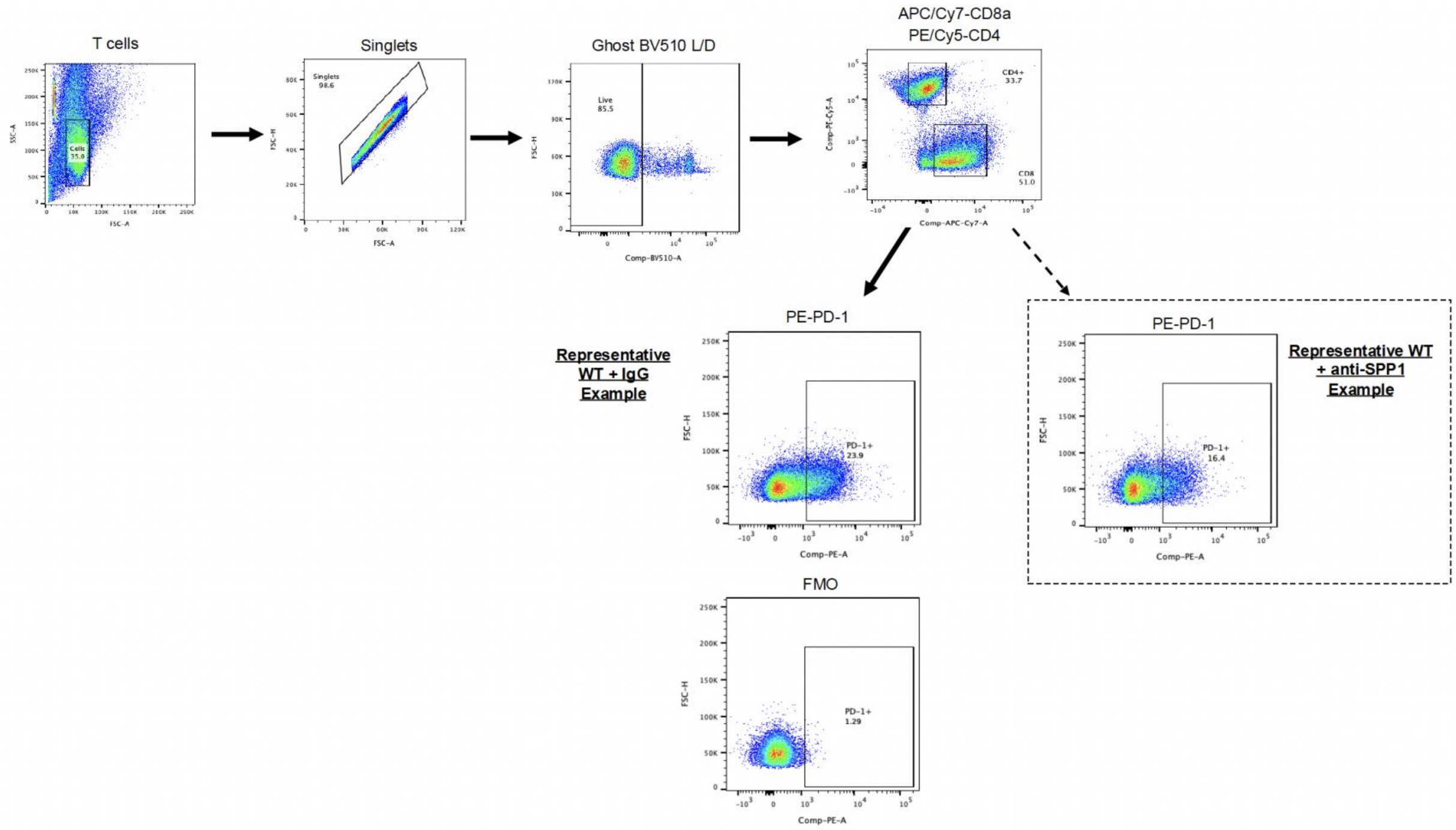

Supplementary Figure S2: Gating strategy for Fig. 4(G), T-cell/BMDM co-culture. T-cell population isolated from debris into singlets, live cells, and PD-1+ CD8+ CD4- live single cells as the final gate. FMO gates for PD-1 are represented alongside representative samples across conditions tested.

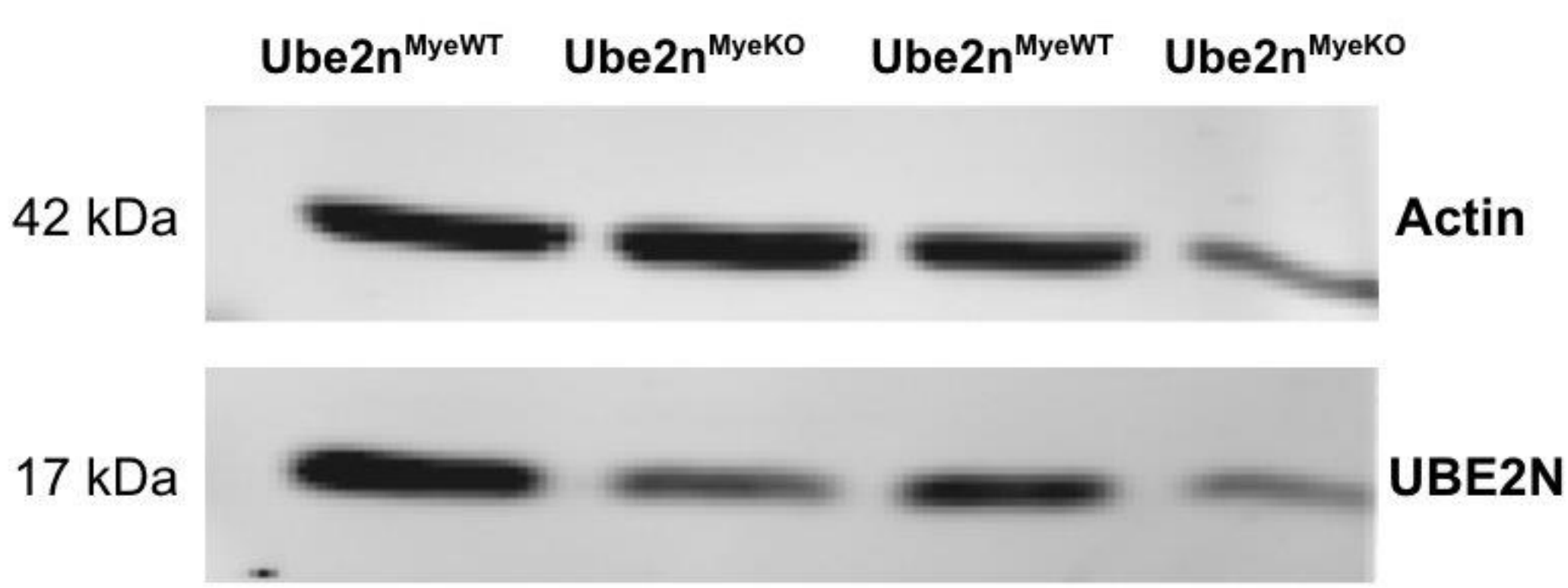

Supplementary Figure S3: UBE2N protein is reduced in Ube2nMyeKO bone marrow-derived macrophages. Immunoblot of whole-cell lysates from BMDMs generated from *Ube2n*^{MyeWT} and *Ube2n*^{MyeKO} mice probed for UBE2N (17 kDa) and Actin (42 kDa) as a loading control. Lanes 1-2 and 3-4 represent two independent bone marrow preparations. Actin signal is

reduced in lane 4, indicating a lower total protein load in that lane. The load-matched comparison of lanes 1 and 2 therefore provides the clearest assessment of UBE2N reduction. Blots were cropped to the region shown.

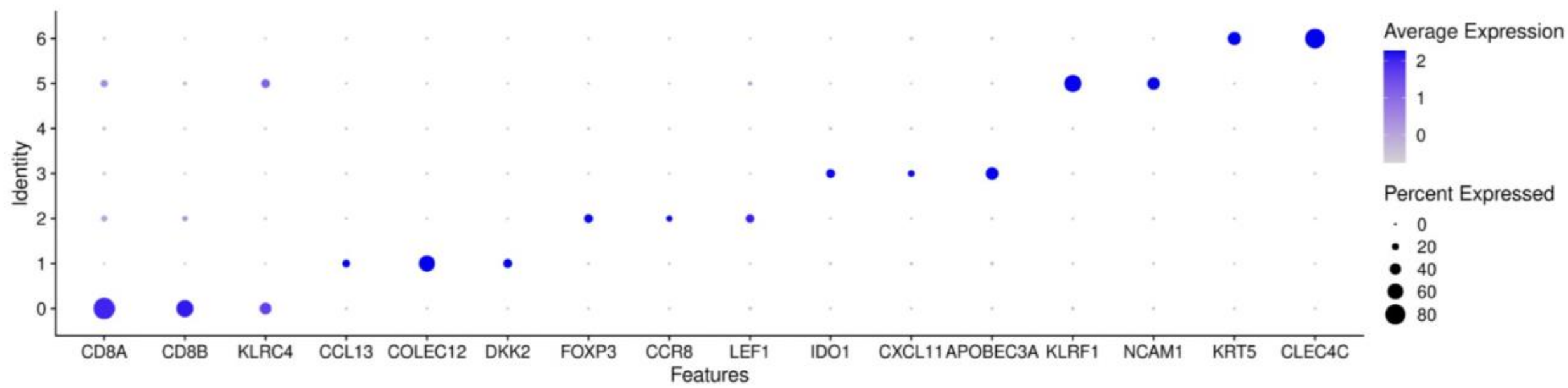

Supplementary Figure S4: Representation of top DEGs used to differentiate G3 sub-clusters. Cluster identities were assigned based on the top 10 DEGs in each unique cluster. Here, a single unique identifier gene with associated expression Z-score is represented to provide context for sub-cluster identity assignments.

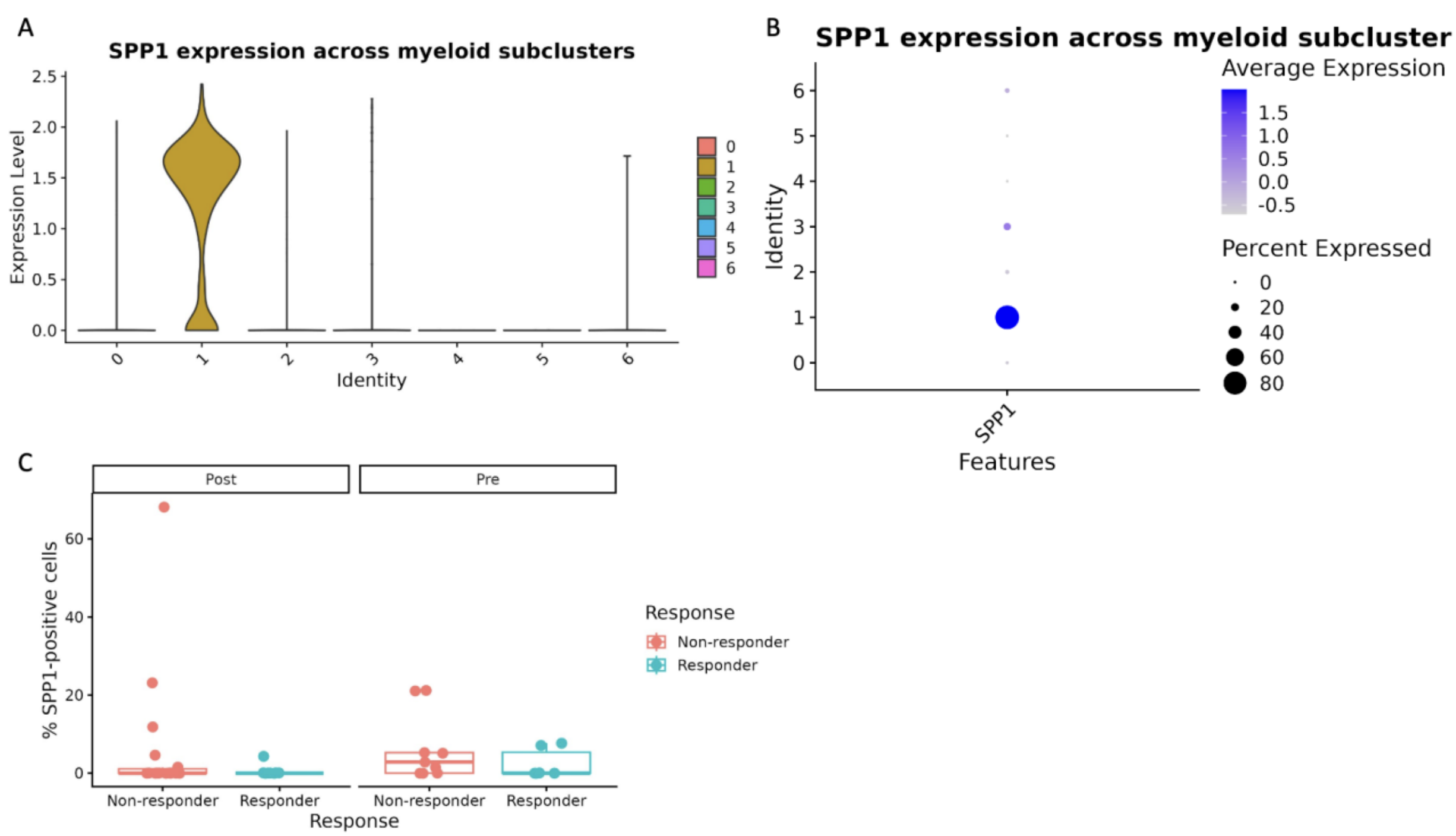

Supplementary Figure S5: SPP1 expression across macrophage sub-clusters and in pre- and post-ICI patients. (A) Violin plot depicting SPP1 expression across all sub-clusters of the monocyte/macrophage population post-ICI. (B) Dot plot representation of what % of cells in

each sub-cluster express SPP1 post-ICI. All data represented in (A) and (B) are from post-ICI patient cohorts. (C) Dot plot representation of % SPP1-positive cells in Pre and Post-ICI patients.

Table S1. RT-PCR primers.

Mouse *Ube2n Forward*	TGAGAGCAACGCCCGTTATTT
Mouse *Ube2n Reverse*	GCCATTGGGTATTCTTCTGGAA
Mouse *Stx5a Forward*	CGGAAACGCTACGGATCTAAG
Mouse *Stx5a Reverse*	CAGGGGACAGAACCTGTGT
Mouse *Spp1 Forward*	AGCAAGAAACTCTTCCAAGCAA
Mouse *Spp1 Reverse*	CTGTCCTGGTATTGAGGGTGG
Mouse *Vegfa Forward*	GAGGTCAAGGCTTTTGAAGGC
Mouse *Vegfa Reverse*	CCGAAAAGCTGTCCCACAAAA
Mouse *Tnfa Forward*	CCCTCACACTCACAAACCAC
Mouse *Tnfa Reverse*	ACAAGGTACAACCCATCGGC
Mouse *Apoe Forward*	CTGACAGGATGCCTAGCCGA
Mouse *Apoe Reverse*	CCTGCTCAGGTTGTTGCTGG
Mouse *Gapdh Forward*	GCACAGTCAAGGCCGAGAAT
Mouse *Gapdh Reverse*	GCCTTCTCCATGGTGGTGA
Mouse Genotyping: *Ube2n wt Forward*	ACACCTTTAATCCCAGCAGAGGCCTAC
Mouse Genotyping: *Ube2n common Reverse*	CTCTGCCCCTCTGCTGATCTTTAACAT
Mouse Genotyping: *Ube2n ko Forward*	CTAAAGCGCATGCTCCAGACTGCCTTG

Table S2. Key resources table.

REAGENT or RESOURCE	SOURCE	IDENTIFIER
**Antibodies**		
Rat IgG	Bio X Cell	Cat#:BE0089; RRID: AB_1107769
FITC anti-mouse CD45	BioLegend	Cat#:103108; Clone#30-F11; RRID: AB_312973
PE anti-mouse/human CD11b	BioLegend	Cat#:101208; Clone#M1/70; RRID: AB_312791
PE anti-mouse CD8a	BioLegend	Cat#:100708; Clone#53-6.7; RRID: AB_312747
FITC anti-mouse CD3e	BioLegend	Cat#:152304; Clone#500A2; RRID: AB_2632667
PE anti-mouse CD279 (PD-1)	BioLegend	Cat#:135205; Clone#29F.1A12; RRID: AB_1877232
PE anti-mouse CD274 (B7-H1, PD-L1)	BioLegend	Cat#:124308; Clone#:10F.9G2; RRID: AB_2073556
AlexaFluor 488 Donkey anti-Rabbit IgG (H+L)	Invitrogen	Cat#:A21206
DAPI	Thermo Scientific	Cat#:62248
APC/Cyanine7 anti-mouse CD8	BioLegend	Cat#:100714; Clone#:53-6.7; RRID: AB_312753
PE-Cy5 anti-mouse CD4	BioLegend	Cat#:100514; Clone#:RM4-5; RRID: AB_312717
FITC anti-mouse I-A/I-E	BioLegend	Cat#:107605; Clone#M5/114.15.2; RRID: AB_313320
Anti-UBE2N/Ubc13	Cell Signaling Technology	Cat#:4919; RRID: AB_2211168
TruStain FcX Rat IgG anti-mouse CD16/CD32	BioLegend	Cat#:101320; Clone#:93; RRID: AB_1574975
Dynabeads™ Mouse T-Activator CD3/CD28	Thermo Fisher	Cat#11456D
Ultra-LEAF™ Purified anti-mouse CD3ε Antibody	BioLegend	Cat#103164; Clone#:30-F1
PerCP/Cyanine5.5 anti-mouse CD223 (LAG-3) Antibody	BioLegend	Cat#:125211
APC anti-mouse CD366 (Tim-3) Antibody RMT3-23	BioLegend	Cat#:119705
Anti-Osteopontin SPP1 Rabbit Monoclonal Antibody	Boster	Cat#:M00634; Clone#CEO-19; UniProt ID#P10451
InVivoMAb anti-mouse/human/rat osteopontin (SPP1)	BioXcell	Cat#BE0382
InVivoMAb mouse IgG1 isotype control	BioXcell	Cat#:BE0083
**Chemicals, peptides, and recombinant proteins**		
4-hydroxytamoxifen	Sigma	Cat#:H7904
DMEM	Thermo Fisher	Cat#:11965092

FBS	R&D	Cat#:S11150H
Antibiotic-Antimycotic	Thermo Fisher	Cat#:15240096
RPMI 1640	Thermo Fisher	Cat#:11875093
Recombinant Mouse M-CSF Protein	R&D	416-ML-010
Recombinant murine IL-2	PeproTech	Cat#:212-12
Recombinant murine IL-4	PeproTech	Cat#:214-14
Recombinant murine IL-13	PeproTech	Cat#:210-13
Recombinant murine IFNy	PeproTech	Cat#:315-05
Matrigel	Corning	Cat#:354234
Ghost Dye™ Violet 510	TONBO	Cat#:13-0870-T500
TRIzol reagent	Invitrogen	Cat#:15596018
2X Universal SYBR Green Fast qPCR Mix	ABclonal	Cat#:RK21203
**Critical commercial assays**		
Pan T-Cell Isolation Kit II	Miltenyi	Cat#:130-095-130
iScript™ Reverse Transcription Supermix System	Bio-Rad	Cat#:1708840
Mouse OPN ELISA Kit	Ray Biotech	Cat#:ELM-OPN-1
Applied Biosystems™ StepOne™ Real-Time PCR System	Thermo Fisher	Cat#:4376357
**Deposited data**		
ScRNAseq data referenced in this study: NCBI, accession # GSE120575		
**Experimental models: Cell lines**		
Yumm1.7	ATCC	Cat#:CRL-3362; RRID: CVCL_JK16
**Experimental models: Organisms/strains**		
Ube2n^{C87S} Mutant and WT bone marrow	Provided by Dr. Daniel Starczynowski	Cincinnati Children's Hospital Medical Center, Cincinnati, OH, USA
Mouse: *LysM^{CreER}*	Jackson Laboratories	Strain # 032291
Rosa^{CreER}Ube2n$^{fl/fl}$	Provided by Dr. Shao-Cong Sun	Chinese Institute for Immunology, Chinese Institutes for Medical Research, Beijing, China
**Software and algorithms**		
ImageJ/Fiji	Schneider et al.; Schindelin et al.	https://imagej.nih.gov/ij/; RRID: SCR_003070
Seurat version 4.1.1	Satija et al.	RRID: SCR_007322
R version 4.0.5		RRID: SCR_001905
SCTransform	Hafemeister & Satija	RRID: SCR_022146
FlowJo version 10.0		https://www.flowjo.com/solutions/flowjo; RRID: SCR_008520
GraphPad Prism version 9.0.0		RRID: SCR_002798
